# Legal Intelligence

**Published:** 1873-09

**Authors:** 


### LEGAL INTELLIGENCE.

### BEFORE JUDGE JOHNSON, SUPERIOR COURT, MONTREAL.

BOWKER *vs.* BEERS.—The parties are both dentists residing here ; and the Plaintiff brings his action against the Defendant for having, with intent to injure the Plaintiff in his character personally and professionally, written and published in the March number of the *Canada Journal of Dental Science*, certain commentaries on another article that had appeared in the January number of the *Canada Medical Journal*, signed by the Plaintiff. The *Canada Journal of Dental Science* is printed at Hamilton, in Ontario, but the publication by Defendant in Montreal is what is complained of in the present case, and it is proved that the *C. J. of Dental Science* was circulated here, and received by five witnesses, and also that the Defendant is one of the editors and publishers of it. This is all there is as to the fact of publication here. What is in issue under the 2nd plea, and under the circumstances, I hold it to be enough.

1st. The Defendant, by his plea, admits that he wrote the article complained of, and said that it was partly provoked and called for by the previous production of Dr. Bowker, to which it was an answer. The subject of this controversy was the use of amalgam by dentists for filling cavities in the teeth, and the Plaintiff commenced the discussion in the *Canada Medical Journal.* It cannot be said that it was not a fit subject for discussion in the interest of dentists and of their customers. The only ground of complaint could be that the discussion was not conducted in a fit and proper manner, that the dispute ceased to be scientific and became personal.

The Plaintiff in the article that called forth the one complained of, by his action had a perfect right to condemn the use of amalgam.

He used that right, but unfortunately did not stop there. After exposing its noxious properties and effects, he says: "The question is often and naturally asked why this amalgam is so generally used by a certain class of dentists." The answer can be found in one or all of the following explanations:

1st. The cheapness of the material.

2nd. The ease and facility with which it is used, for it can be put into the most difficult cavities with as much ease as so much putty or wax.

3rd. It makes up for the want of skill and ability to use something better.

4th. From ignorance or the want of honesty.

The defendant replied to this article in the *Canada Journal of Dental Science* in the same temper. Not content with refuting that part about the amalgam in point of fact, he says: "Dr. Bowker, you are an imposter; you yourself use this very article which you condemn in others." Now this is a libel like the first; but the first was a libel on the profession, while the second is one on Dr. Bowker. If he had considered himself libelled as a member of the profession, Beers might have sued the author, but he did not do so, but he libels again. It is to be observed that he is charged with a *wanton* and *malicious* libel. Now it cannot be considered such, but was written under provocation, and not wanton or maliciously. This will go in mitigation of damages, which I have placed very low. Judgment for 50 shillings damages and costs of an action of the lowest class in the Superior Court. *A. & W. Robertson* for Plaintiff; *Carter & Keller* for Defendant.—*Montreal Herald.*

